# Case Series of Post-COVID-19 Mucormycosis in Serbia/Rhino-Orbital-Cerebral Form: Surgical and Medical Treatment

**DOI:** 10.1155/crdi/8385268

**Published:** 2025-08-04

**Authors:** Nikola Mikovic, Ana Durkovic, Novica Boricic, Marko Lazic, Zoran Jezdic, Vitomir Konstantinovic

**Affiliations:** ^1^Clinic for Maxillofacial Surgery, University of Belgrade Faculty of Dental Medicine, Dr. Subotica 4, Belgrade 11000, Serbia; ^2^Pathology Clinic, University of Belgrade Faculty of Medicine Belgrade, Dr. Subotica 1, Belgrade 11000, Serbia

**Keywords:** case series, mucormycosis, post-COVID-19, rhino-orbital-cerebral, surgical

## Abstract

**Introduction:** Mucormycosis is a rare but serious infection caused by fungi called mucormycetes. It is life-threatening, highly aggressive angioinvasive infection, which mainly affects immunocompromised people.

**Methods and Results:** During 2021–early 2023, at the Clinic for Maxillofacial Surgery, Faculty of Dental Medicine, University of Belgrade, Serbia (the “Clinic”), medical team (the “Team”) treated five patients, with a rhino-orbital-cerebral form of mucormycosis. All five patients had recently recovered from COVID-19 infection prior to detection of mucormycosis. All of them spent a considerable amount of time (on average 1 month of hospitalization) at COVID-19 specialized hospitals. The Team treated these patients following the guidelines for screening diagnosis and management of mucormycosis at the time of the COVID-19 pandemic (COVID-19 patient guidelines/Indian Council of Medical Research, published in May 2021 approved by WHO). Treatment included several phases, out of which the Team was responsible for and carried out early diagnosis and surgical intervention phase, while colleagues from other clinics assisted in other phases of treatment/management of mucormycosis.

**Conclusion:** The goal of this paper is to present five patients diagnosed with mucormycosis, with a special focus on two patients who, due to their condition, received surgical intervention as part of their antifungal treatment.

## 1. Introduction

Mucormycosis is a rare, noncontagious fungal infection with a global incidence rate of 0.005–1.7 per million population [1]. Rhino-orbital-cerebral mucormycosis is the most common type, accounting for 30%–50% of all cases, and it poses a life-threatening risk, often spreading to the orbits and brain [2].

Recent data suggest that India has the highest prevalence of mucormycosis, with estimates indicating a rate nearly 70 times higher than the global average [3]. Notably, the reporting of COVID-19-associated mucormycotic infections has increased during the COVID-19 pandemic, primarily in India, followed by USA, Pakistan, France, Iran, and other countries [4]. Patients with uncontrolled diabetes mellitus (DM) and prolonged neutropenia or lymphopenia (CD4) are at a higher risk of developing mucormycosis [5]. The use of immunosuppressive therapy like corticosteroids, prolonged mechanical ventilation with oxygen therapy, and high doses of antibiotics in COVID-19 treatment may contribute to opportunistic infections such as mucormycosis [6, 7]. The clinical presentation of rhino-orbital-cerebral mucormycosis resembles complicated sinusitis, with symptoms such as nasal blockage, crusting, facial pain, tooth loss, proptosis, ptosis, chemosis, and various neurological signs if there is intracranial involvement [8]. Timely diagnosis and treatment are crucial, as delayed intervention can lead to rapid disease progression, with reported mortality rates ranging from 50% to 80% [9]. Although some studies have shown high mortality rates of 25%–60% [10], this particular study presents a high recovery rate.

This article aims to present five patients diagnosed with post-COVID-19 mucormycosis and to review the literature regarding the prevention, diagnosis, and management of this fatal infection. The study emphasizes a case series of two patients who received complete surgical and medicament treatment.

## 2. Cases Presentation

### 2.1. Methods

We share our experience in managing five cases of mucormycosis infections spanning from 2021 to early 2023. Our hospital stands out for having the patients of rhino-orbital-cerebral forms of mucormycosis treated nationwide.

Out of the five patients, four were male and one was female, with an average age of 65 years. Patients were referred to our clinic directly following recovery from COVID-19 infection. All patients received treatment at COVID-19 hospitals, prompted by either mild or severe COVID-19 infection. They were administered oxygen therapy, from mechanical ventilation along with prolonged and high-dose antibiotic therapy. Additionally, three patients received high-dose glucocorticoid therapy, while two were subjected to immunosuppressive therapy.

All patients exhibited symptoms including headache, nasal congestion, and loose teeth upon presentation. Clinical examinations revealed palatal and alveolar ridge eschars in the majority of cases, alongside varying degrees of buccal swelling. One patient presented with an oroantral fistula, while another exhibited ophthalmoplegia accompanied by double vision. All patients presented with comorbidities, with two cases classified as severe. One patient had hematological malignancy (non-Hodgkin lymphoma), while another had metastatic colorectal carcinoma. Additionally, two patients had chronic kidney disease, and three had previously diagnosed and treated DM.

Given the patients' history of COVID-19 infection, suspicion for mucormycosis was raised during clinical examination, prompting biopsy procedures in all cases for confirmation.

**Figure 1:**
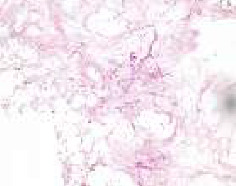
Histopathological specimen (numerous hyphae are seen, some of them sectioned longitudinally, some of them transversely. HE x200).

A biopsy was performed under local anesthesia. In contrast to conventional procedures in surgical pathology, which generally involve the examination of viable tissue, the diagnosis of mucormycosis in all patients presented here was established through the detection of fungal hyphae within necrotic tissue. Hematoxylin and Eosin (H&E) staining is sufficient in diagnosis where abundant hyphae are present. In cases where hyphae are sparse, additional histochemical staining, such as Grocott's methenamine silver (GMS) stain, is necessary to enhance fungal visibility. The fungal elements typically appear as thick, nonseptated, ribbon-like hyphae with right-angle branching, or as circular profiles in transverse sections (Figure 1) [11]. In rare cases, the fungus is found in viable tissue in granulomas, in patients with better immunity.

Three patients were confirmed to have rhino-orbital forms of mucormycosis via CT imaging. However, one patient initially diagnosed with rhino-orbital mucormycosis progressed to rhino-orbital-cerebral form following primary surgical intervention. Notably, one patient did not undergo CT confirmation of rhino-orbital involvement, as they declined further diagnostic procedures and medical treatment. Clinical characteristics, radiological findings, and treatment are shown in Table 1.

One patient declined further treatment upon receiving biopsy results. Two patients succumbed shortly after the initiation of antifungal therapy: one due to complications associated with COVID-19 and the other due to the progression of metastatic rectal carcinoma and its complications. Among the five patients referred to our hospital, only two underwent surgical intervention in conjunction with antifungal therapy.

## 3. Results

### 3.1. Case 1

A 58-year-old male with a history of hypertension, an artificial aortic valve implant, and chronic kidney disease has been admitted to the Clinic with intense pain in the upper jaw, numbness, fetor ex ore, and nasal congestion. Upon physical examination, a tearing right eye and a swollen lower eyelid were observed, as well as necrotic intraoral presentation of the upper jaw with brown and blackish discoloration of the palate, mucosa missing at the alveolar ridge, and loosening of maxillary teeth on the right side. The patient's medical records revealed that he had severe complications during his COVID-19 hospitalization which lasted for 41 days, including a Hartmann resection procedure due to bleeding from the rectosigmoid part of the colon. During this hospital treatment, he was administered high-dose corticosteroid and antibiotic therapy (i.v. azithromycin, ceftriaxone, and prednisolone (Table 1)) for 10 days to treat severe pneumonia. During this treatment, due to the deterioration of his condition, the patient underwent an intensive care regimen, including intravenous steroids and oxygen therapy mechanical ventilation (CT score: 16/25) for 31 days with antibiotics (including linezolid, meropenem, and metronidazole (Table 1)).

**Figure 2:**
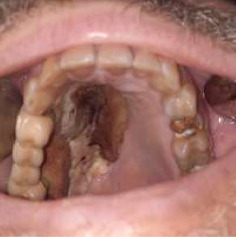
Intraoral clinical presentation (see eschar), case 1.

**Figure 3:**
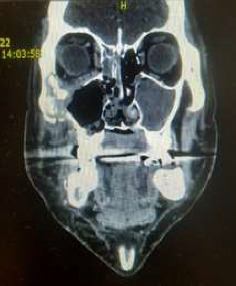
Coronal CT scan view (see bone presentation), case 1.

**Figure 4:**
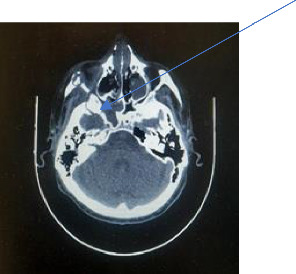
Axial CT scan (see suspected intracranial involvement), case 1.

After his discharge from the COVID-19 hospital, the CT of the splanchnocranium identified changes in the maxillary sinuses, right ethmoid, zygomatic bone, severe necrosis of the lateral walls and floor of the nose, partial necrosis of the nasal septum, damage to the right orbital floor, and medial wall (see Table 1, patient number 1) and suspected intracranial involvement indicated by infiltration of the sphenoid sinus walls and from the superior orbital fissure (please see Figures 2, 3, and 4). A biopsy was performed, and the histopathology results confirmed mucormycotic-infected tissue. The Team decided that the patient had to receive a 28-day course of antifungal therapy with liposomal amphotericin B 5 mg/kg (see Table 1, patient number 1) in the intensive care unit and subsequently undergo surgical intervention. A right total maxillectomy was performed along with a partial maxillectomy on the left side, accompanied by partial resection of the right nasal and zygomatic bone. Immediate surgical obturators were employed to address functional, hygienic, and psychological concerns.

Follow-up CT after 6 weeks showed progression of the disease with erosion of temporal and sphenoid bone with intracranial involvement along superior orbital fissure. The patient undertook a neurosurgical procedure to manage the intracranial involvement. The affected mucormycotic zone was removed, followed by another dose of antifungal treatment in the intensive care unit for four more weeks (parenteral administered amphotericin B 3 mg/kg/day due to suboptimal kidney function), then due to intracranial involvement 6 mg/kg for 15 days (435 mg + 261 mg + 522 mg). However, the administration of antifungal medication was intermittent due to the patient's overall health condition and suboptimal kidney function (G3). Six months after the end of the treatment, the MRI of the endocranium and CT of the splanchnocranium exhibited no radiological signs of mucormycosis. Therefore, his health condition at that time allowed for a definitive surgical reconstruction of resected facial bones to be planned.

### 3.2. Case 2

A 59-year-old former smoker male with a history of hypertension was hospitalized for severe bilateral COVID-19 pneumonia for one and a half months in a specialized COVID-19 hospital. During hospitalization, the patient received systemic steroids (methylprednisolone) for a total of 27 days, along with high-dose antibiotic therapy for 45 days, including combinations of ceftriaxone, vancomycin, meropenem, levofloxacin, bactrim, metronidazole, and amikacin (Table 1, Patient 2). Oxygen therapy was administered for varying durations. During COVID-19 hospitalization, patient complaints on tooth pain and intraoral swelling. After dental examination, and consultation of specialist, oral surgeon suggested teeth extraction. Upper jaw pain and facial edema on the right side led to the extraction of teeth 14 and 15.

**Figure 5:**
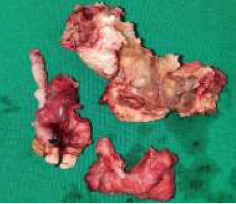
Surgical specimen, Case 2.

One month after COVID-19 hospitalization, the patient was referred to the clinic due to worsening pain in the upper jaw, buccal edema enlargement, exposed bone, and foul oral smell. Physical examination revealed buccal edema, exposed brownish and yellow-colored bone of the alveolar process and tuber of the maxilla, and an exposed maxillary sinus and a part of the hard palate. CT scan of the splanchnocranium was performed and indicated the involvement of the maxilla on both sides, the hard palate, and nasal bones, with suspected infiltration of the right orbit along the infraorbital canal. Additionally, destruction of the sphenoid sinus walls, the right greater wing of the sphenoid, and lateral lamina of the pterygoid process were observed. Biopsy confirmed mucormycosis, and the patient underwent surgical removal of necrotic and affected bone and soft tissue. A right subtotal maxillectomy was performed, along with a partial maxillectomy on the left side. Part of the affected nasal bone and all three nasal conchae were also surgically removed on the right side (Figure 5) (see Table 1, patient number 2). Immediate surgical obturators were utilized to cover the defect. After 20 days of postoperative care, the patient was released and transferred to another clinic for antifungal therapy in the intensive care unit. Liposomal amphotericin B was administered at a dose of 5 mg/kg/day for four weeks, followed by 3 mg/kg/day for two weeks (480 mg + 288 mg) [12]. Antifungal therapy was temporarily interrupted due to suboptimal kidney function.

Once the control CT showed no radiological signs of mucormycosis, antifungal therapy was discontinued, and the patient was discharged from the hospital in a stable condition. Monthly regular follow-ups at the clinic revealed no signs of necrotic or affected residual bone and no further disease progression. After 1 year of follow-ups, the patient was disease-free.

## 4. Discussion/Conclusion

Mucormycosis, a rare but life-threatening fungal infection caused by mucormycete group of organisms, has garnered significant attention during the COVID-19 pandemic [3]. This study presented the first five cases of post-COVID-19 mucormycosis in Serbia, with a particular focus on two patients who received surgical intervention as part of their antifungal treatment. The findings of this study gain a comprehensive understanding of the prevention, diagnosis, and management of this fatal infection.

The COVID-19 pandemic has presented a unique challenge, with an increasing number of mucormycosis cases reported in COVID-19 patients. This association has been primarily observed in countries such as India, USA, Pakistan, France, and Iran [3]. This association has been primarily observed in countries such as India, USA, Pakistan, France, and Iran [4]. The use of corticosteroids, other immunosuppressive therapy, prolonged mechanical ventilation, and high-dose antibiotics in COVID-19 treatment may contribute to the increased susceptibility to opportunistic infections like mucormycosis [6, 7]. Furthermore, individuals with uncontrolled DM and compromised immune systems are at a higher risk of developing mucormycosis, and the presence of these comorbidities may be more prevalent in COVID-19 patients [5].

DM, recognized as a well-established risk factor for mucormycosis, is associated with elevated rates of morbidity and mortality in COVID-19 [9, 10]. Consistent with the pathophysiology of other severe infections, patients affected by COVID-19 are predisposed to diabetic ketoacidosis (DKA). Existing evidence supports the notion that SARS-CoV-1 induces damage to pancreatic islets, leading to the onset of acute diabetes and subsequent DKA. This phenomenon may serve as a plausible explanation for the observed “diabetogenic state” in SARS-CoV-2 infection [13, 14]. DM, recognized as a well-established risk factor for mucormycosis, is associated with elevated rates of morbidity and mortality in COVID-19 [13, 14]. Consistent with the pathophysiology of other severe infections, patients affected by COVID-19 are predisposed to DKA. Existing evidence supports the notion that SARS-CoV-1 induces damage to pancreatic islets, leading to the onset of acute diabetes and subsequent DKA. This phenomenon may serve as a plausible explanation for the observed “diabetogenic state” in SARS-CoV-2 infection [15, 16]. The significant acute cortisol stress response is elicited by COVID-19, compounded by concurrent glucocorticoid therapy. These elevated serum cortisol levels exacerbate glycemic control in both diabetic and nondiabetic patients. This exacerbation of hyperglycemia heightens the risk of fungal infection [17]. Out of five patients mentioned, three presented with a prior diagnosis of diabetes. Among the two surgically treated patients depicted, neither had a history of diabetes prior to nor during their COVID-19 hospitalization.

Four of our patients received high-dose glucocorticoid therapy during COVID-19 infection, in order to treat and prevent acute respiratory distress syndrome (ARDS).

Glucocorticoids, through their dose-dependent immunosuppressive effect, inhibit the ability and phagocytic functions of phagocytes, the main host defense mechanism against mucormycetes, thereby reducing their capacity to migrate to infected tissue and eliminate the microorganism [18]. To prevent secondary bacterial infection, they all received high doses of broad-spectrum antibiotics which furthermore increased the risk of developing opportunistic fungal infections such as mucormycosis [19].

The clinical presentation of rhino-orbital-cerebral mucormycosis often mimics severe sinusitis, with symptoms such as nasal congestion, facial pain, proptosis, and cranial nerve palsies if there is intracranial involvement [8]. In a systematic review by Jeong et al., the overall mortality rate of mucormycosis was reported to be approximately 50%, highlighting the severity of this infection [20]. However, it is important to note that the present study demonstrated a high recovery rate, indicating successful management strategies employed by the medical team.

In this study, the Team followed a screening, diagnosis, and management guidelines for mucormycosis during the COVID-19 pandemic, as recommended by the Indian Council of Medical Research and approved by the World Health Organization [12]. Early diagnosis is crucial for timely intervention, and this case presentation focused on the role of surgical intervention as part of the antifungal treatment. Surgical resection of necrotic and affected tissue is a cornerstone in the management of mucormycosis, as it reduces the fungal burden and allows for better penetration of antifungal agents [5, 21–23].

Moreover, antifungal therapy plays a vital role in the management of mucormycosis. Liposomal amphotericin B is considered the first-line antifungal agent, and prolonged administration is often required [12]. The administration of antifungal medications should be tailored based on patient characteristics, including renal function, as these agents can have nephrotoxic effects [1]. The combination of surgical intervention and appropriate antifungal therapy can significantly improve patient outcomes and increase the likelihood of complete eradication of the infection, as observed in the cases discussed in this study.

The emergence of post-COVID-19 mucormycosis, particularly the rhino-orbital-cerebral form, underscores the critical need for early diagnosis and prompt, aggressive treatment. Our case series highlights the importance of a multidisciplinary approach involving maxillofacial surgeons, infectious disease specialists, and neurosurgeons to manage this life-threatening infection effectively. Despite the high morbidity and mortality associated with mucormycosis, our findings demonstrate that significant good clinical outcomes can be achieved with timely intervention. Notably, even in cases with intracranial involvement, the combination of surgical debridement and antifungal therapy led to successful patient outcomes. This emphasizes the importance of early clinical diagnosis and the necessity of adhering to established treatment protocols to significantly improve patient survival rates. Continued vigilance and research are essential to better understand the pathogenesis, risk factors, and optimal management strategies for mucormycosis in the context of COVID-19 and beyond.

## Figures and Tables

**Table 1:** Characteristics of COVID-19-associated mucormycosis cases: Clinical presentation, diagnosis, medical and surgical intervention, and outcomes.

Case no	1	2	3	4	5
Age	58	59	51	79	73
Sex	M	M	F	M	M
Indication of admission	Mucor	Suspicious osteomyelitis of the upper jaw	COVID-19	Mucor	Mucor
Prior steroid therapy	Prednisone 80 mg daily, (14 days) Methylprednisolone 64 mg daily, (21 days)	Methylprednisolone 32 mg daily (during the hospitalization period at COVID hospital)	No	Prednisone 40 mg (14 days)	Methylprednisolone 32 mg daily (14 days)
Prior antibiotic therapy	I.V. Azithromycin 1500 mg, daily (7 days) I.V. Ceftriaxone 2 g daily (14 days) I.V. Meropenem 2 g, daily (7 days)	I.V. Meropenem 3 g daily (7 days) I.V. Levofloxacine 1 g daily (14 days) I.V. Bactrim 960 mg daily (7 days) I.V. Metronidazole 1.5 g daily (7 days) I.V. Amikacin 1 g (7 days) I.V. Vancomycin 1 g (7 days)	I.V. Meropenem 1 g daily (7 days)	I.V. Vancomycin 1 g (treatment duration not available from medical records)	I.V. Ceftriaxone 2 g (treatment duration not available from medical records) I.V. Azithromycin 500 mg (treatment duration not available from medical records)
Known/new DM	No	No	Yes	Yes	Yes
Comorbidities	- HTN - CKD - AF - ITP - Previous colon resection with colostomy - Aortic valve replacement	- HTN	- NHL	- CKD - AF - HTN - Metastatic rectal cancer - Pulmonary fibrosis	- HTN
COVID-19 hospital treatment duration	41 days	45 days	32 days	29 days	33 days
Symptoms/clinical findings	- Headache, nasal congestion, facial numbness - Palatal and alveolar ridge eschar - Loose teeth of the upper jaw	- Buccal swelling, upper jaw pain - Palatal and alveolar ridge eschar, oroantral fistula	- Headache, facial swelling, double vision - Chemosis, periorbital swelling, ophthalmoplegia, palatal eschar, buccal skin induration, and discoloration	- Upper jaw pain, oral fetor - Periorbital and buccal swelling, alveolar ridge eschar, loose teeth of the upper jaw	- Tooth pain, nasal congestion - Loose teeth, buccal induration, palatal eschar
Tissue fungal	Yes (no growth)	Yes (no growth)	No	No	No
Histopathology	Yes	Yes	Yes	Yes	Yes
Description of radiological finding	Contrast CT: Total opacification of the left maxillary sinus, right ethmoidal cells right sphenoid sinus, bulky inferior rectal and inferior oblique ocular muscle on the right side, proptosis of the right eye, bony erosion of right zygomatic bone, all maxillary sinus walls Follow-up CT after 6 weeks: Progression of the disease with the erosion of temporal and sphenoid bone with intracranial involvement along superior orbital fissure	Contrast CT: Opacification of the right maxillary sinus with mucosal thickening of both maxillary sinus and nasal cavity. Bony erosions of the maxilla on both sides, hard palate, nasal bones, sphenoid sinus walls, and right greater wing of a sphenoid and lateral lamina of the pterygoid process. Bulky right lateral pterygoid muscle. Mild right eye proptosis.	Complete opacification and mucosal thickening of left maxillary and ethmoid sinus. Refraction of left maxillary sinus bone. Involvement of left zygomatic bone.	Contrast CT: Mucosal thickening of right maxillary and sphenoid sinus, linear hyperdensities in right maxillary sinus associated with early bone change	Not done
Treatment	Surgical intervention + antifungal therapy + maintenance therapy	Surgical intervention + antifungal therapy	Antifungal therapy	Antifungal therapy	None
Surgical intervention	Total right maxillectomy and partial left maxillectomy, partial resection of the right zygomatic and right nasal bone, partial ethmoidectomy, removal of orbital floor and pterygoid on the right side	Right subtotal maxillectomy, partial maxillectomy on the left side, removal of the right nasal bone, and ipsilateral nasal turbinates	Not done	Not done	Patient refused treatment
Antifungal therapy	I.V. Amphotericin 5 mg/kg/day 4 weeks followed by 3 mg/kg/day 4 weeks due to cranial involvement	I.V. Amphotericin B 5 mg/kg/day 6 weeks with therapy interruption	I.V. Amphotericin B 5 mg/kg/day 3 weeks treatment was interrupted due to patient's death	I.V. Amphotericin 3 mg/kg/day 6 weeks	Patient refused treatment
Maintenance therapy	Oral posaconazole 6 months	N/A	None	None	Patient refused treatment
Treatment duration	9 months	3 months			
Outcome	Discharged without radiographic or clinical signs of the disease	Discharged without radiographic or clinical signs of the disease	Died 3 weeks after treatment initiation	Died 1 year after diagnosis and treatment	N/A
Cause of death	—	—	Septic shock due to COVID-19	Advanced metastatic rectal carcinoma	N/A

*Note:* F, female; HTN, hypertension; IV, intravenous; M, male.

Abbreviations: AF, atrial fibrillation; CKD, chronic kidney diseases; CT, computerized tomography; DM, diabetes mellitus; ITP, idiopathic thrombocytopenic purpura; NA, not applicable; NHL, non-Hodgkin lymphoma; PO, per oral.

## Data Availability

The data that support the findings of this study are available on request from the corresponding author. The data are not publicly available due to privacy or ethical restrictions.
